# Association between the triglyceride to high-density lipoprotein cholesterol ratio and 90-day post-stroke depression in acute ischemic stroke

**DOI:** 10.3389/fpsyt.2026.1827410

**Published:** 2026-07-24

**Authors:** Xiangqi Kong, Yuning Wang, Haobo Wang, Xinyue Yuan, Mina Zhao, Wei Jing

**Affiliations:** Third Hospital of Shanxi Medical University, Shanxi Bethune Hospital, Shanxi Academy of Medical Sciences, Tongji Shanxi Hospital, Taiyuan, China

**Keywords:** AIS, HDL, insulin resistance, post-stroke depression, TG

## Abstract

**Background:**

Post-stroke depression (PSD) commonly complicates acute ischemic stroke (AIS), with insulin resistance (IR)-mediated metabolic disturbances proposed as an underlying pathophysiological link. The triglyceride to high-density lipoprotein cholesterol ratio (TG/HDL)—a validated, cost-effective surrogate for IR—has preliminary associations with depressive symptoms. However, the relationship between TG/HDL and the risk of PSD in patients with AIS remains unclear.

**Methods:**

A cohort of 519 AIS patients was retrospectively analyzed (October 2023–December 2024). The primary outcome was 90-day PSD, diagnosed via the HAMD-24 (score > 7). Multivariable logistic regression assessed the independent association between admission TG/HDL and PSD. Nonlinear associations were assessed using restricted cubic splines (RCS).

**Results:**

Thirty-three point nine percent of patients developed PSD. The TG/HDL ratio was an independent predictor of PSD (Model 3: OR = 1.5634, 95% CI: 1.0097–2.4281, *P* = 0.0452). RCS analysis suggested a potential non-linear relationship, and a two-piecewise logistic regression model identified a candidate inflection point. However, the wide confidence intervals in the higher exposure range indicate substantial uncertainty, and this finding should be regarded as exploratory.

**Conclusions:**

Admission TG/HDL ratio was independently associated with 90-day PSD, suggesting that it may serve as a convenient metabolic marker for identifying patients at higher risk of developing PSD. The observed non-linear pattern requires cautious interpretation and validation in larger prospective cohorts.

## 1 Introduction

Post-stroke depression (PSD) represents a prevalent neuropsychiatric sequela of acute ischemic stroke (AIS), affecting approximately 30–50% of patients within three months of onset, with symptoms ranging in severity (1, 2). Beyond severely impairing patients’ neurological recovery and functional status, PSD also significantly increases the likelihood of recurrent stroke and mortality, emerging as a critical barrier to stroke rehabilitation and secondary prevention (3, 4). Despite its substantial clinical significance, the pathogenesis of PSD remains incompletely elucidated, and reliable early predictive markers are lacking in clinical practice (5). Emerging evidence suggests that metabolic dysregulation, particularly insulin resistance (IR), may play an important role in the development of both cerebrovascular disease and depressive disorders, making it a potential mechanistic link underlying PSD.

In recent years, insulin resistance (IR) and the metabolic disturbances it mediates have attracted increasing attention as a potential “pathophysiological bridge” linking cerebrovascular disease and affective disorders (6, 7). IR has emerged as an independent determinant of AIS, contributing to stroke onset and progression by exacerbating endothelial dysfunction and accelerating atherosclerotic processes (8, 9). Concurrently, the central nervous system metabolic imbalance, excessive activation of oxidative stress, and neuroinflammatory cascade reactions induced by IR may directly interfere with cerebral mood regulation pathways, thereby participating in the pathophysiological process of post-stroke depression (10–12). Accordingly, the identification of accessible biomarkers that reliably reflect IR status may enable early recognition of high-risk PSD patients.

Among the various surrogate markers proposed for assessing IR, the triglyceride-to-high-density lipoprotein cholesterol ratio (TG/HDL) has attracted considerable attention. Owing to its simplicity, accessibility, and low cost, TG/HDL has been widely validated as a practical surrogate marker of IR in both cardiovascular and metabolic research settings (13–16). Existing evidence indicates that a higher TG/HDL is linked to poor prognoses in various cardiovascular and metabolic conditions, including coronary heart disease, type 2 diabetes, and metabolic syndrome (17–19). More importantly, the pathophysiological implications of this ratio may extend beyond the metabolic domain to the field of mental health (20). Epidemiological studies have provided preliminary evidence linking dyslipidemia to depressive symptoms: a large-scale cross-sectional study revealed a J-shaped dose-response association between TG/HDL ratio levels and depressive symptoms, suggesting a pathophysiological basis for their non-linear association (21).

However, longitudinal evidence linking the TG/HDL ratio to PSD risk in AIS patients remains extremely limited. Although a small-sample study reported higher TG/HDL may be associated with mood disorders one month post-stroke (22), the reliability and generalizability of its conclusions are questionable due to insufficient sample size, short follow-up duration, and failure to adequately control for key confounding factors such as cognitive impairment. More importantly, the potential nonlinear relationship between TG/HDL and PSD has not been systematically investigated.

Given the paucity of longitudinal evidence regarding the association of TG/HDL with PSD following AIS, and the absence of prediction models based on this indicator, the present research sought to: (1) examine the independent association between admission TG/HDL and the development of PSD within 90 days in AIS patients; and (2) further evaluate the potential nonlinear relationship between TG/HDL and PSD risk using restricted cubic spline (RCS) analysis. Through this investigation, we sought to provide new metabolic evidence to facilitate the early identification of patients at high risk of PSD.

## 2 Methods

### 2.1 Participants

Patients diagnosed with AIS at the Department of Neurology, Shanxi Bethune Hospital, from October 2023 to December 2024 were retrospectively analyzed. In accordance with the definition established by the World Health Organization, patients with AIS were enrolled, and all diagnoses were verified by MRI or brain CT in hospitalized patients.

Eligibility criteria included CT/MRI-confirmed ischemic stroke within 72 h of onset per the 2018 Chinese Guidelines for AIS, first-ever stroke, age ≥18 years, and complete clinical data.

Exclusion criteria included non-ischemic cerebrovascular disease, prior psychiatric illness, inability to complete assessments, severe systemic comorbidities, chronic inflammatory disease, incomplete data, and loss to follow-up.

**Figure 1:**
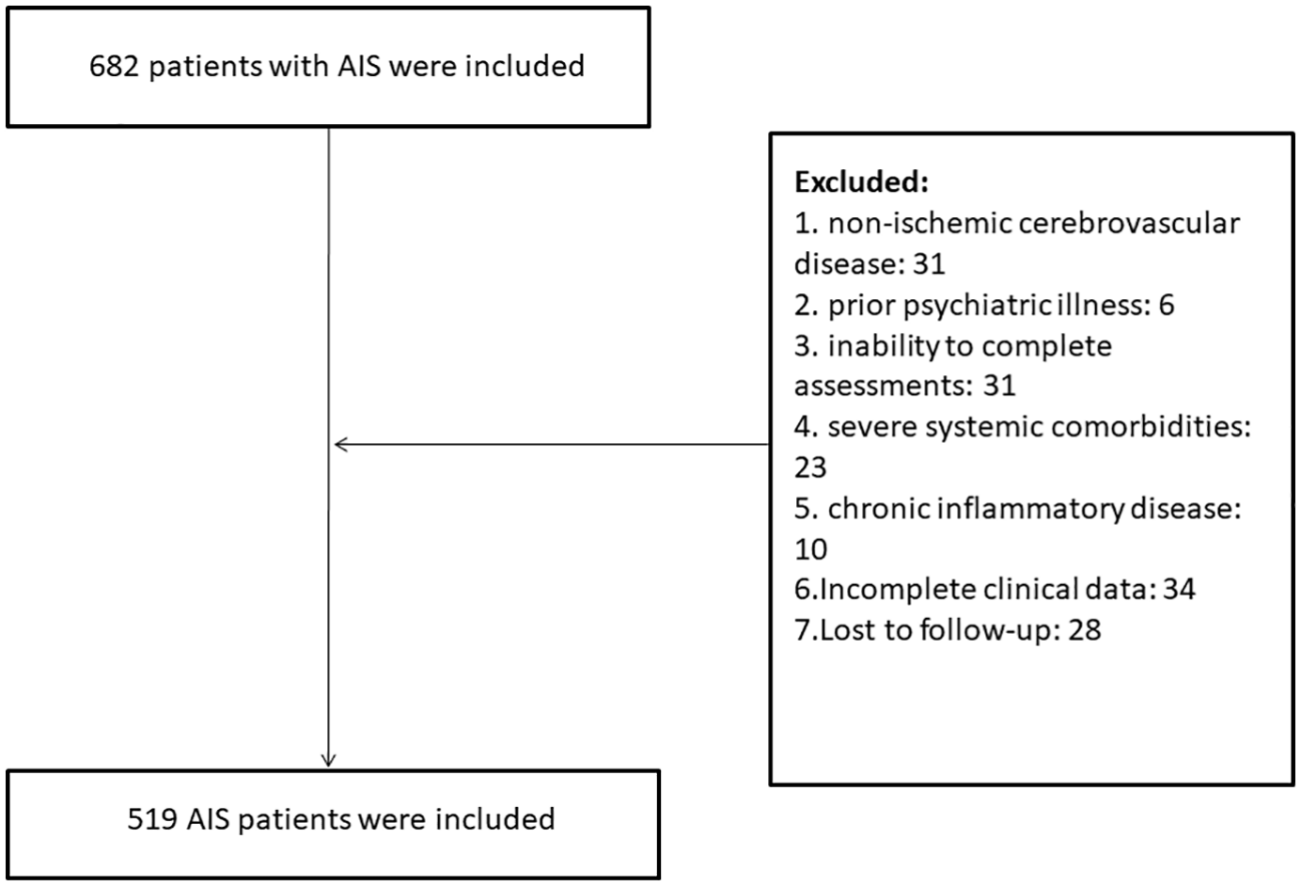
Flowchart of participant selection.

The analysis ultimately involved 519 eligible participants (**Figure 1**).

### 2.2 Data acquisition

For all patients, clinical and demographic information was documented, comprising body mass index (BMI), Montreal Cognitive Assessment (MoCA) score, and National Institutes of Health Stroke Scale (NIHSS) score, as well as age, sex, educational level, infarct location, responsible lesion, histories of hypertension (HTN), diabetes mellitus (DM), and coronary artery disease (CAD), together with smoking status and alcohol consumption. Stroke severity was assessed using the NIHSS score within 24 hours of admission, and cognitive function was evaluated using the MoCA score after clinical stabilization during hospitalization.

For laboratory assessments, fasting venous blood samples were obtained within 24 hours of admission prior to initiation of metabolic therapies. Samples were collected using standardized protocols, including 2 mL in EDTA-K_2_ anticoagulant tubes for complete blood count measurements (white blood cell count (WBC), monocyte count (Mon), neutrophil count (Neu), lymphocyte count (Lym), and platelet count (PLT)) using a fully automated hematology analyzer (Beckman DXH800), and 5 mL in non-anticoagulant tubes for serum biochemical analyses of homocysteine (Hcy), low-density lipoprotein cholesterol (LDL), total cholesterol (TC), high-density lipoprotein cholesterol (HDL), triglycerides (TG), serum uric acid (UA) after centrifugation. D-dimer concentrations were assessed in citrated plasma (1:9) with a Werfen ACL-TOP 750 analyzer.

The TG/HDL ratio was defined as the value of TG divided by HDL.

### 2.3 Study outcomes

PSD was defined as a HAMD-24 score >7 at 90 days post-stroke, consistent with operational definitions used in prior stroke studies (23). Although structured psychiatric diagnostic interviews were not performed, two trained clinicians independently assessed depressive symptoms using the Chinese version of HAMD-24 while blinded to clinical and laboratory data, including TG/HDL levels. The absence of a formal diagnostic interview may introduce potential misclassification. Formal inter-rater reliability statistics of HAMD-24 were not calculated and reported. However, the HAMD-24 has demonstrated excellent reliability and validity in previous studies (24).

### 2.4 Statistical analysis

Baseline characteristics were compared according to the presence or absence of PSD. For continuous variables, the Shapiro-Wilk test was used to verify the normality of data distribution: normally distributed variables were presented as mean ± standard deviation (SD), while non-normally distributed variables were expressed as median (25th, 75th percentiles). Differences between the two groups were compared using the independent samples t-test (for normally distributed data) or the Mann-Whitney U test (for non-normally distributed data). For categorical variables, data were summarized as frequencies and percentages, and intergroup differences were analyzed using the chi-square test. Cases with missing values in key exposure, outcome, or covariate variables were excluded from the final analytic dataset. No multiple imputation or other missing data handling techniques were performed.

Multivariable logistic regression analysis was performed to assess the association between TG/HDL and PSD. Confounding variables were adjusted for based on results of univariate analyses (P < 0.05) and clinical relevance. Three regression models were constructed: Model 1: No adjustment for confounding variables; Model 2: Adjusted for sex, smoking status, and alcohol consumption; Model 3: Further adjusted for NIHSS score, MoCA score, Neu, and D-dimer level.

In sensitivity analyses, a test for linear trend was conducted using TG/HDL quartiles as the independent variable to evaluate the robustness of the association. Restricted cubic spline (RCS) regression was used to examine potential non-linear associations between TG/HDL and PSD, with 4 knots placed at the 5th, 35th, 65th, and 95th percentiles of the TG/HDL distribution. The median value was used as the reference category. Non-linearity was assessed using a likelihood ratio test comparing spline and linear models. A piecewise linear regression model was performed for exploratory purposes to further evaluate potential non-linear patterns. The cutoff value of 1.80 was derived from exploratory examination of the spline curve and was not intended to represent a predefined or biologically meaningful threshold. Additionally, subgroup and interaction analyses were used to examine the relationship between TG/HDL and PSD across subgroups defined by sex, smoking, alcohol consumption, HTN, and DM.

These analyses were exploratory in nature, and no adjustment for multiple comparisons was applied. Statistical analyses were conducted in R Studio (version 4.4.2), with a two-sided P < 0.05 indicating statistical significance.

## 3 Results

### 3.1 Baseline characteristics of participants

Among 519 included patients, 343 were classified as non-PSD and 176 as PSD (**Table 1**). Sex, smoking, drinking, MoCA, NIHSS, neutrophil count, D-dimer, and TG/HDL ratio differed significantly between the groups (P < 0.05). Specifically, the PSD group had a higher proportion of males, higher rates of drinking and smoking, significantly elevated D-dimer levels, NIHSS scores, and TG/HDL ratios, whereas MoCA scores were notably lower in this group.

**Table 1:** Baselines characteristics of participants.

Characteristic	Overall N = 519^1^	Non-PSD N = 343^1^	PSD N = 176^1^	p-value^2^
Sex				<0.001
Female	159 (31%)	129 (38%)	30 (17%)	
Male	360 (69%)	214 (62%)	146 (83%)	
Education				0.813
illiteracy	85 (16%)	58 (17%)	27 (15%)	
Primary schhool	183 (35%)	116 (34%)	67 (38%)	
junior high school	209 (40%)	141 (41%)	68 (39%)	
senior high school and above	42 (8.1%)	28 (8.2%)	14 (8.0%)	
Smoking				<0.001
No	214 (41%)	159 (46%)	55 (31%)	
Yes	305 (59%)	184 (54%)	121 (69%)	
Drinking				0.003
No	263 (51%)	190 (55%)	73 (41%)	
Yes	256 (49%)	153 (45%)	103 (59%)	
HTN				0.736
No	210 (40%)	137 (40%)	73 (41%)	
Yes	309 (60%)	206 (60%)	103 (59%)	
DM				0.578
No	362 (70%)	242 (71%)	120 (68%)	
Yes	157 (30%)	101 (29%)	56 (32%)	
CAD				0.228
No	476 (92%)	311 (91%)	165 (94%)	
Yes	43 (8.3%)	32 (9.3%)	11 (6.3%)	
LesionLocation				0.137
Basal ganglia or lateral ventricles	227 (44%)	142 (41%)	85 (48%)	
Brain stem or Cerebellum	147 (28%)	103 (30%)	44 (25%)	
Thalamus	14 (2.7%)	12 (3.5%)	2 (1.1%)	
cerebral lobe	34 (6.6%)	26 (7.6%)	8 (4.5%)	
Multiple infarction	97 (19%)	60 (17%)	37 (21%)	
StrokeLocation				0.412
Left	324 (62%)	209 (61%)	115 (65%)	
Right	173 (33%)	117 (34%)	56 (32%)	
Both	22 (4.2%)	17 (5.0%)	5 (2.8%)	
NIHSS	2.00 (1.00, 4.00)	2.00 (1.00, 3.00)	4.00 (3.00, 5.00)	<0.001
Age (years)	63.00 (55.00, 70.00)	62.00 (55.00, 70.00)	63.00 (56.00, 70.00)	0.988
MoCA	25.00 (21.00, 26.00)	25.00 (24.00, 26.00)	21.00 (19.00, 24.00)	<0.001
BMI	22.36 (18.60, 27.09)	22.15 (18.30, 26.78)	23.17 (19.46, 27.81)	0.074
WBC (×10^9/L)	6.40 (5.30, 7.70)	6.40 (5.30, 7.70)	6.40 (5.53, 7.95)	0.272
Lym (×10^9/L)	2.54 (1.99, 3.40)	2.58 (1.99, 3.40)	2.53 (1.99, 3.37)	0.604
Mon (×10^9/L)	0.53 (0.44, 0.65)	0.52 (0.43, 0.65)	0.55 (0.46, 0.65)	0.238
Neu (×10^9/L)	5.94 (4.77, 6.86)	6.02 (5.16, 6.89)	5.64 (3.35, 6.83)	0.017
PLT (×10^9/L)	238.96 (194.88, 284.69)	236.22 (194.28, 284.20)	241.92 (196.62, 287.48)	0.327
D-dimer (ng/mL)	121.00 (76.00, 243.00)	112.00 (69.00, 192.00)	175.00 (93.00, 347.50)	<0.001
TG (mmol/L)	1.47 (1.22, 1.74)	1.47 (1.17, 1.82)	1.47 (1.23, 1.72)	0.645
TC (mmol/L)	4.16 (3.48, 4.88)	4.16 (3.51, 4.98)	4.15 (3.47, 4.68)	0.377
LDL (mmol/L)	2.67 (2.22, 3.20)	2.61 (2.19, 3.27)	2.70 (2.27, 3.11)	0.364
Hcy (umol/L)	17.00 (12.90, 26.50)	16.20 (12.90, 25.80)	17.85 (13.20, 27.20)	0.306
UA (mmol/L)	298.40 (244.40, 381.70)	294.90 (240.30, 378.40)	305.60 (254.35, 387.05)	0.148
HDL (mmol/L)	0.93 (0.81, 1.09)	0.99 (0.87, 1.16)	0.83 (0.75, 0.93)	<0.001
TG/HDL	1.56 (1.21, 2.00)	1.47 (1.10, 1.90)	1.75 (1.38, 2.15)	<0.001

^1^ n (%); Median (Q1, Q3).

^2^ Pearson’s Chi-squared test; Fisher’s exact test; Wilcoxon rank sum test.

HTN, hypertension; DM, diabetes mellitus; CAD, Coronary Artery Disease; WBC, white blood cell; Lym, lymphocyte; Mon, monocyte; Neu, neutrophil; PLT, platelet counts; TG, triglycerides; TC, total cholesterol; LDL, low-density lipoprotein; HDL, high-density lipoprotein; Hcy, homocysteine; UA, uric acid.

### 3.2 Association between TG/HDL and post-stroke depression

Univariate regression analysis demonstrated positive associations between sex, alcohol consumption, smoking status, MoCA score, NIHSS score, neutrophil count, D-dimer level, HDL level, TG/HDL, and PSD risk (see **Supplementary Table 1**). Multivariable logistic regression analysis (see **Table 2**) further confirmed a significant positive correlation between TG/HDL and PSD. In Model 1 (unadjusted), each 1-unit increase in the TG/HDL ratio was associated with a 107.72% increase in the risk of PSD (OR = 2.0772, 95% CI: 1.5246–2.8527, P < 0.0001). This association remained significant in Model 2, after adjusting for sex, smoking status, and alcohol consumption (OR = 1.9794, 95% CI: 1.4413–2.7403, P < 0.0001). Even after further adjustment for additional confounding factors in Model 3, the association retained statistical significance (OR = 1.5634, 95% CI: 1.0097–2.4281, P = 0.0452).

**Table 2:** Multivariable logistic regression analysis of the association between the TG/HDL and PSD.

Exposures	Model 1 OR (95% CI), *P*-value	Model 2 OR (95% CI), *P*-value	Model 3 OR (95% CI), *P*-value
TG/HDL	2.0772 (1.5246, 2.8527), <0.0001	1.9794 (1.4413, 2.7403), <0.0001	1.5634 (1.0097, 2.4281), 0.0452
Quartiles			
Q1	Reference	Reference	Reference
Q2	1.5914 (0.8997, 2.8479), 0.1128	1.5740 (0.8803, 2.8463), 0.1285	1.5872 (0.7146, 3.5737), 0.2587
Q3	2.8800 (1.6687, 5.0697), 0.0002	3.0065 (1.7192, 5.3640), 0.0001	2.5275 (1.1850, 5.5297), 0.0179
Q4	3.3239 (1.9329, 5.8385), <0.0001	3.1166 (1.7905, 5.5359), 0.0001	2.1736 (1.0207, 4.7314), 0.0465
P for trend	<0.0001	<0.0001	0.0268

Model 1 = no covariates were adjusted.

Model 2 = Model 1 + sex, smoking, drinking, were adjusted.

Model 3 = Model 2 +  NIHSS, MoCA, Neu, D-dimer, were adjusted.

Additionally, when TG/HDL was analyzed by quartiles, in the Model 3, the highest quartile (Q4) was significantly associated with an increased PSD risk (OR = 2.1736, 95% CI: 1.0207–4.7314, P = 0.0465). The test for linear trend yielded a P-value of 0.0268, indicating that higher TG/HDL quartiles were associated with increased PSD risk.

### 3.3 Assessment of the non-linear relationship

**Figure 2:**
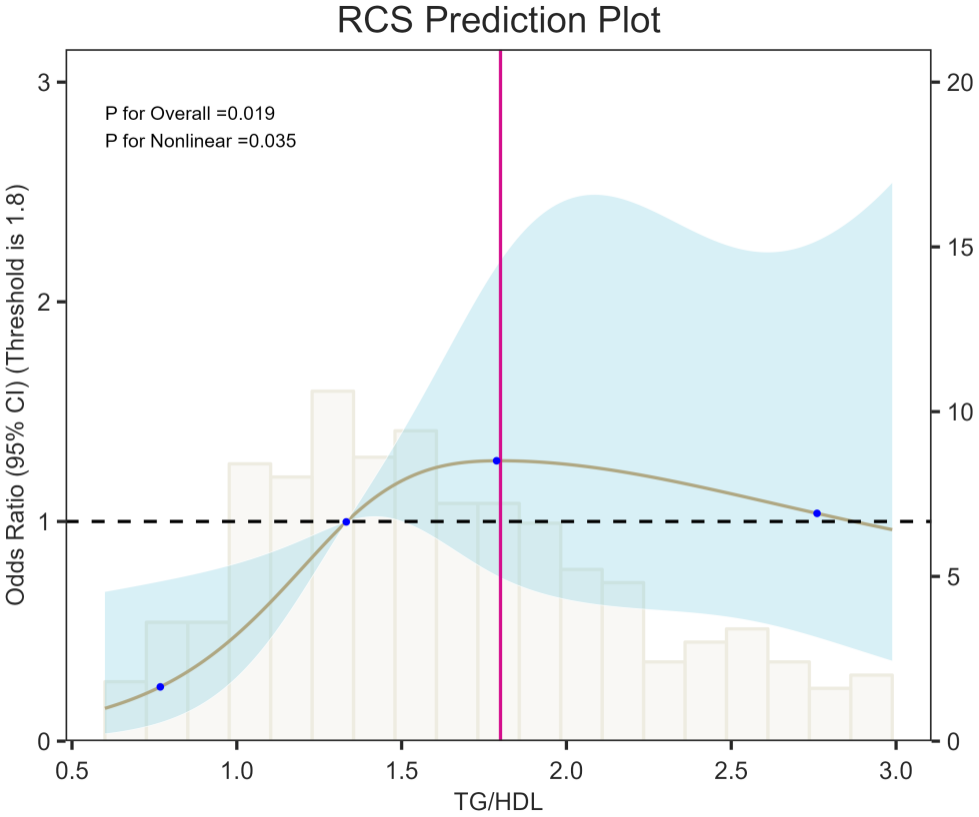
RCS showing the association between TG/HDL and 90-day PSD.

RCS analysis (**Figure 2**) indicated that the risk of PSD at 90 days increased with higher TG/HDL levels, showing a significant overall association (P for overall = 0.019). Further testing suggested a potential non-linear relationship between TG/HDL and PSD (P for non-linearity = 0.035). However, the shape of this association should be interpreted with caution.

A two-piecewise logistic regression model identified a potential inflection point at TG/HDL = 1.80 (**Table 3**). When TG/HDL was below 1.80, higher levels were associated with an increased risk of PSD (OR = 4.3901, P = 0.0019). When TG/HDL exceeded 1.80, the association was not statistically significant (OR = 0.5954, P = 0.2531), although the estimate was imprecise, with wide confidence intervals.

**Table 3:** Threshold effect analysis for associations of TG/HDL with PSD.

Exposures	TG/HDL
Linear effect model	
OR, (95%CI), P-value	1.5634 (1.0097, 2.4281), 0.0452
Non-linear model	
Inflection point (K)	1.80
OR, (95%CI), P-value (< K)	4.3901 (1.7549, 11.4600), 0.0019
OR, (95%CI), P-value (≥ K)	0.5954 (0.2388, 1.4231), 0.2531
Likelihood Ratio Test, P-value	0.0112

Model 3 covariates were adjusted.

However, given the moderate sample size and limited data density in the higher exposure range, these findings should be interpreted as exploratory rather than evidence of a definitive threshold effect. The likelihood ratio test (P = 0.0112) suggests that a non-linear model may better fit the data, but no stable plateau or biologically established cut-off can be confirmed.

In summary, higher TG/HDL levels were associated with an increased risk of PSD, while the observed non-linear pattern requires cautious interpretation and external validation.

### 3.4 Subgroup analyses

**Figure 3:**
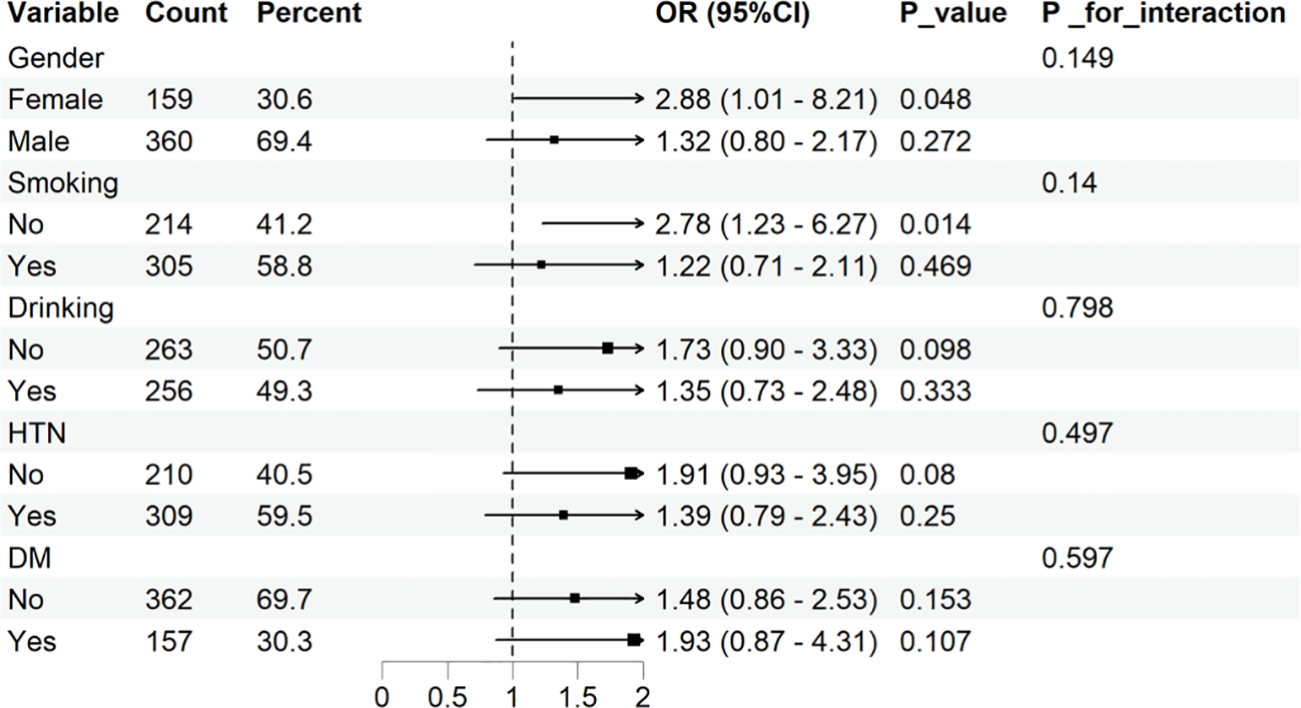
Subgroup analysis for the association between TG/HDL and 90-day PSD.

Subgroup analyses were performed stratified by sex, smoking, alcohol consumption, HTN, and DM. As shown in **Figure 3**, no significant interaction was observed between these subgroups and the TG/HDL ratio (all P-values for interaction > 0.05).

### 3.5 Sensitivity analyses

To assess the robustness of the findings under a more stringent definition of PSD, a sensitivity analysis was performed using HAMD-24 >10 as the outcome. The association between TG/HDL and PSD remained generally consistent with the primary analysis. In the fully adjusted model, participants in the third and fourth TG/HDL quartiles remained at significantly higher risk of PSD compared with the lowest quartile, and a significant dose–response trend was preserved (**Supplementary Table 2**). Given that neutrophil count and D-dimer may lie on the potential causal pathway between metabolic dysfunction and PSD, we conducted a sensitivity analysis excluding these variables to evaluate the robustness of the main findings. The results remained consistent with the primary analysis after excluding neutrophil count and D-dimer, suggesting that the observed association was not materially influenced by potential overadjustment for intermediate inflammatory or coagulation-related variables. (**Supplementary Table 3**). To further examine the individual contributions of lipid parameters to the observed association between the TG/HDL ratio and PSD, we performed separate analyses for TG, HDL, and TG/HDL. As shown in **Supplementary Figure 1**, TG levels did not differ significantly between PSD and non-PSD groups (P = 0.65), whereas HDL was markedly lower in the PSD group (P < 0.001), confirming that the elevated TG/HDL ratio in PSD patients is primarily driven by low HDL rather than elevated TG. **Supplementary Table 4** presents multivariable logistic regression models for TG, HDL, and TG/HDL separately. After full adjustment (Model 3), HDL remained strongly and independently associated with PSD (OR = 0.0221, 95% CI: 0.0097–0.1007, P = 0.0001), while TG alone showed no significant association (OR = 0.6921, 95% CI: 0.3696–1.2364, P = 0.2309). The TG/HDL ratio retained its independent significance (OR = 1.5634, 95% CI: 1.0097–2.4281, P = 0.0452), suggesting that the composite marker captures a broader metabolic risk profile than its individual components alone.

## 4 Discussion

This is the first large retrospective study to examine the association between admission TG/HDL ratio and 90-day PSD in patients with AIS. We found that TG/HDL was independently associated with PSD, and that the relationship may be non-linear.

The findings of this study corroborate and extend the limited exploratory evidence available to date. A prior small-sample study reported an association between elevated TG/HDL ratios and mood disorders at 1 month post-stroke (22); however, its conclusions were constrained by small sample size and inadequate control of confounding variables. Notably, our research confirmed the independent association between TG/HDL ratios and mid-term PSD (90 days post-stroke) by leveraging a larger sample size, a longer follow-up duration, and rigorous adjustment for key confounding factors—including neurological deficit severity (NIHSS score), cognitive status (MoCA score), and inflammatory markers. These results provide more robust longitudinal evidence supporting the potential value of TG/HDL as a metabolic indicator for early PSD risk assessment in AIS patients.

Restricted cubic spline analysis suggested a potential non-linear association between TG/HDL and PSD. However, the shape of this relationship should be interpreted with caution. A two-piecewise regression model indicated a possible inflection point at TG/HDL = 1.80. Below this value, higher TG/HDL was associated with increased PSD risk, whereas above this level, the association was attenuated and no longer statistically significant. However, given the moderate sample size and limited data density in the upper exposure range, this pattern may reflect statistical instability rather than a definitive biological threshold. Therefore, no firm plateau or cutoff can be established based on the current data. Compared with prior studies in the general population (21), which reported a J-shaped association between TG/HDL and depressive symptoms, our findings suggest a potentially different pattern in AIS patients. This discrepancy may reflect differences in population characteristics, underlying vascular burden, and acute neurobiological changes following stroke. However, such comparisons remain speculative and require further validation in independent cohorts.

This non-linear association may reflect multiple interconnected mechanisms, though these remain speculative given our observational design. Insulin resistance—for which TG/HDL has been used as a surrogate marker in some populations—may contribute to PSD risk through neuroinflammation (25–27). cerebrovascular endothelial dysfunction (28–30), oxidative stress, and downregulation of BDNF expression (31–33). In addition, the pattern observed in our cohort suggests a prominent role for low HDL, which may independently promote neuroinflammation, impair antioxidant defenses, and compromise endothelial repair and nitric oxide bioavailability (34–37). These pathways are not mutually exclusive and may act synergistically, with the TG/HDL ratio capturing their cumulative metabolic burden. However, direct measurements of inflammatory, oxidative, and metabolic biomarkers are needed to validate these mechanisms in future studies.

It is noteworthy that while only HDL-C showed a significant between-group difference among the individual lipid parameters, the composite TG/HDL ratio exhibited a substantially stronger association with PSD. This pattern suggests that the ratio captures a synergistic metabolic disturbance—combining relatively low HDL-C with a concurrent trend of elevated TG—that may better reflect underlying systemic lipid dysregulation than any single parameter alone. This phenomenon aligns with the concept that composite indices integrate subtle, coordinated changes in lipid metabolism that may not reach statistical significance when analyzed separately, and supports the use of ratio-based markers over isolated lipid fractions in prognostic studies (38).

Several strengths of this study merit consideration. The 90-day follow-up data allowed us to capture clinically meaningful post-stroke outcomes, while the hierarchical adjustment for stroke severity, cognitive function, inflammatory markers, and coagulation parameters across three regression models enhanced the robustness of our findings despite the retrospective design. Notably, the use of RCS analysis enabled us to characterize a non-linear association between the TG/HDL ratio and PSD risk, providing a preliminary reference for risk stratification in future hypothesis-driven studies. Furthermore, as a routine, low-cost laboratory test available in both tertiary and primary care settings, the TG/HDL ratio offers high accessibility and reproducibility, supporting its potential utility as a practical screening tool in post-stroke populations. Additionally, the use of real-world clinical data, drawn from routine practice rather than controlled trial conditions, may enhance the generalizability of our findings to typical post-stroke populations.

Several limitations should be acknowledged. First, the retrospective single-center design limits causal inference, and residual confounding cannot be excluded. Although we adjusted for multiple demographic, clinical, and stroke-related variables, including NIHSS, lesion location, and major vascular risk factors, several important factors—such as pre-stroke depressive symptoms or psychiatric history, antidepressant use, rehabilitation participation, social support, stroke subtype, and lesion volume—were not available and may have influenced the observed associations. While patients with known psychiatric illness were excluded, baseline depressive symptoms were not systematically assessed, and thus residual confounding from undiagnosed pre-stroke depression remains possible. Second, lipid parameters were measured only once during the acute phase, without repeated monitoring during follow-up. Acute inflammatory stress may also distort baseline lipid profiles, potentially affecting the accuracy of risk estimation. Third, the relatively limited sample size—particularly in the higher TG/HDL exposure range—may have reduced the precision of the non-linear and threshold analyses, and the identified inflection point should therefore be regarded as exploratory. Given these limitations, our findings should be interpreted as hypothesis-generating rather than conclusive. Large-sample prospective studies with serial lipid monitoring and comprehensive confounder adjustment are warranted to further validate the clinical utility of the TG/HDL ratio in PSD risk stratification.

## 5 Conclusion

This study demonstrates that a higher admission TG/HDL is independently associated with an increased risk of 90-day PSD in patients with AIS. A potential non-linear relationship was also suggested, though the limited sample size in the higher exposure range precludes confirmation of a stable inflection point. These findings suggest that TG/HDL may serve as a convenient metabolic indicator for identifying AIS patients at higher risk of PSD. Patients with elevated TG/HDL levels may warrant closer emotional monitoring and metabolic assessment, but such strategies require prospective validation. Future multicenter prospective studies with larger sample sizes and direct measurements of metabolic biomarkers are needed to further validate the clinical utility of TG/HDL and to clarify its role in post-stroke risk stratification.

## Data Availability

The original contributions presented in the study are included in the article/**Supplementary Material**. Further inquiries can be directed to the corresponding author.
