# Supplementary material for: Association between the triglyceride to high-density lipoprotein cholesterol ratio and 90-day post-stroke depression in acute ischemic stroke
Source: Front Psychiatry. 2026 Jul 24;17:1827410. doi: 10.3389/fpsyt.2026.1827410 (PMC13447493; doi:10.3389/fpsyt.2026.1827410)
Supplement: Supplementary file 1 [file Supplementaryfile1.docx]

目录

[◼	**Table S1 Univariate logistic regression analysis**	2](#_Toc232809042)

[◼	**Table S2** **Multivariable Logistic Regression Analysis of the Association Between TG/HDL and 90-day PSD (HMHD>10)**.	4](#_Toc232809043)

[◼	**Table S3. Sensitivity analysis of the association between TG/HDL and 90-day post-stroke depression after excluding potential mediators (neutrophil count and D-dimer)**.	5](#_Toc232809044)

[◼	**TableS4 Multivariable Logistic Regression Analysis of the Association Between the TG, HDL,TG/HDL and PSD.**	6](#_Toc232809045)

[◼	**Figure S1. Box plots showing TG, HDL, and TG/HDL ratio distributions by PSD status**	7](#_Toc232809046)

- **Table S1 Univariate logistic regression analysis**

| **Characteristic** | **OR**^1^ | **95% CI**^1^ | **p-value** |
| --- | --- | --- | --- |
| NIHSS | 2.146 | 1.861, 2.505 | <0.001 |
| Age | 0.9977 | 0.9810, 1.015 | 0.785 |
| MoCA | 0.5883 | 0.5323, 0.6455 | <0.001 |
| BMI | 1.023 | 0.9962, 1.052 | 0.092 |
| WBC | 1.062 | 0.9914, 1.138 | 0.085 |
| Lym | 0.8945 | 0.7458, 1.069 | 0.223 |
| Mon | 1.927 | 0.6419, 5.797 | 0.241 |
| Neu | 0.8626 | 0.7829, 0.9498 | 0.003 |
| PLT | 1.002 | 0.9991, 1.004 | 0.218 |
| D-dimer | 1.001 | 1.000, 1.001 | 0.007 |
| TG | 0.7331 | 0.4862, 1.087 | 0.129 |
| TC | 0.8945 | 0.7473, 1.067 | 0.218 |
| LDL | 1.067 | 0.8475, 1.342 | 0.581 |
| HDL | 0.0073 | 0.0020, 0.0237 | <0.001 |
| UA | 1.001 | 0.9989, 1.003 | 0.436 |
| Hcy | 1.001 | 0.9901, 1.011 | 0.877 |
| TG/HDL | 2.077 | 1.525, 2.853 | <0.001 |
| Sex |  |  |  |
| Female | — | — |  |
| Male | 2.934 | 1.893, 4.663 | <0.001 |
| education |  |  |  |
| illiteracy | — | — |  |
| Primary schhool | 1.241 | 0.7226, 2.163 | 0.439 |
| junior high school | 1.036 | 0.6071, 1.795 | 0.898 |
| senior high school and above | 1.074 | 0.4812, 2.344 | 0.859 |
| Smoking |  |  |  |
| No | — | — |  |
| Yes | 1.901 | 1.301, 2.801 | 0.001 |
| Drinking |  |  |  |
| No | — | — |  |
| Yes | 1.752 | 1.215, 2.537 | 0.003 |
| HTN |  |  |  |
| No | — | — |  |
| Yes | 0.9384 | 0.6489, 1.360 | 0.736 |
| DM |  |  |  |
| No | — | — |  |
| Yes | 1.118 | 0.7523, 1.653 | 0.578 |
| CAD |  |  |  |
| No | — | — |  |
| Yes | 0.6479 | 0.3051, 1.281 | 0.231 |
| LesionLocation |  |  |  |
| Basal ganglia or lateral ventricles | — | — |  |
| Brain stem or Cerebellum | 0.7136 | 0.4557, 1.108 | 0.136 |
| Thalamus | 0.2784 | 0.0427, 1.053 | 0.099 |
| cerebral lobe | 0.5140 | 0.2095, 1.140 | 0.119 |
| Multiple infarction | 1.030 | 0.6281, 1.677 | 0.905 |
| StrokeLocation |  |  |  |
| Left | — | — |  |
| Right | 0.8699 | 0.5860, 1.283 | 0.485 |
| Both | 0.5345 | 0.1721, 1.391 | 0.230 |

- **Table S2** **Multivariable Logistic Regression Analysis of the Association Between TG/HDL and 90-day PSD (HMHD>10)**.

| **Exposures** | **Model1**  **OR(95%CI),*P*-value** | **Model2**  **OR(95%CI),*P*-value** | **Model3**  **OR(95%CI),*P*-value** |
| --- | --- | --- | --- |
| TG/HDL | 2.0562(1.5014, 2.8366),<0.0001 | 1.9335(1.4135,  2.7201), 0.0001 | 1.5332(0.9991, 2.3590),0.0505 |
| Quartiles |  |  |  |
| Q1 | Reference | Reference | Reference |
| Q2 | 1.9878(1.0944, 3.6865),0.0260 | 1.9737(1.0731, 3.7038),0.0308 | 2.1852(0.9783, 5.0037),0.0594 |
| Q3 | 3.2851(1.8499, 6.0002),0.0001 | 3.4764(1.9278, 6.4478),0.0001 | 2.9978(1.3921, 6.6693),0.0058 |
| Q4 | 3.5727(2.0182,  6.5113), <0.0001 | 3.3489(1.8661,  6.1800), 0.0001 | 2.4052(1.1241,  5.2967),0.0259 |
| P for trend | <0.0001 | <0.0001 | 0.0264 |

*Model 1 = no covariates were adjusted.

Model 2 = Model 1 + sex, smoking, drinking, were adjusted.

Model 3 = Model 2 +  NIHSS, MoCA, Neu, D-dimer, were adjusted.

- **Table S3. Sensitivity analysis of the association between TG/HDL and 90-day post-stroke depression after excluding potential mediators (neutrophil count and D-dimer)**.

| **Exposures** | **Model1**  **OR(95%CI),*P*-value** | **Model2**  **OR(95%CI),*P*-value** | **Model3**  **OR(95%CI),*P*-value** |
| --- | --- | --- | --- |
| TG/HDL | 2.0772(1.5246, 2.8527),<0.0001 | 1.9794(1.4413,  2.7403), <0.0001 | 1.5737(1.0233, 2.4278),0.0391 |
| Quartiles |  |  |  |
| Q1 | Reference | Reference | Reference |
| Q2 | 1.5914(0.8997, 2.8479),0.1128 | 1.5740(0.8803, 2.8463),0.1285 | 1.7205(0.7843, 3.8294),0.1781 |
| Q3 | 2.8800(1.6687, 5.0697),0.0002 | 3.0065(1.7192, 5.3640),0.0001 | 2.5187(1.1927, 5.4522),0.0168 |
| Q4 | 3.3239(1.9329,  5.8385), <0.0001 | 3.1166(1.7905,  5.5359), 0.0001 | 2.3535(1.1239,  5.0462),0.0250 |
| P for trend | <0.0001 | <0.0001 | 0.0173 |

*Model 1 = no covariates were adjusted.

Model 2 = Model 1 + sex, smoking, drinking, were adjusted.

Model 3 = Model 2 +  NIHSS, MoCA were adjusted.

- **TableS4 Multivariable Logistic Regression Analysis of the Association Between the TG, HDL,TG/HDL and PSD.**

| **Exposures** | **Model1**  **OR(95%CI),*P*-value** | **Model2**  **OR(95%CI),*P*-value** | **Model3**  **OR(95%CI),*P*-value** |
| --- | --- | --- | --- |
| TG | 0.7331(0.4862, 1.0872),0.1295 | 0.7352(0.4874, 1.0918), 0.1335 | 0.6921(0.3696, 1.2364),0.2309 |
| HDL | 0.0073(0.0020, 0.0237), <0.0001 | 0.0079(0.0021, 0.0265), <0.0001 | 0.0221(0.0097, 0.1007),0.0001 |
| TG/HDL | 2.0772(1.5246, 2.8527),<0.0001 | 1.9794(1.4413, 2.7403), <0.0001 | 1.5634(1.0097, 2.4281),0.0452 |

*Model 1 = no covariates were adjusted.

Model 2 = Model 1 + sex, smoking, drinking, were adjusted.

Model 3 = Model 2 +  NIHSS, MoCA, Neu, D-dimer, were adjusted.

- **Figure S1. Box plots showing TG, HDL, and TG/HDL ratio distributions by PSD status**
